# A New Online Mental Health Training Program for Workplace Managers: Pre-Post Pilot Study Assessing Feasibility, Usability, and Possible Effectiveness

**DOI:** 10.2196/10517

**Published:** 2018-07-03

**Authors:** Aimée Gayed, Anthony D LaMontagne, Allison Milner, Mark Deady, Rafael A Calvo, Helen Christensen, Arnstein Mykletun, Nick Glozier, Samuel B Harvey

**Affiliations:** ^1^ School of Psychiatry Faculty of Medicine University of New South Wales Randwick Australia; ^2^ School of Population and Global Health The University of Melbourne Melbourne Australia; ^3^ Centre for Population Health Research Deakin University Geelong Australia; ^4^ Black Dog Institute Faculty of Medicine University of New South Wales Sydney Australia; ^5^ School of Electrical and Information Engineering University of Sydney Sydney Australia; ^6^ Department of Mental Health and Suicide Norwegian Institute of Public Health Oslo Norway; ^7^ Department of Community Medicine, University of Tromsø Tromsø Norway; ^8^ Centre for Work and Mental Health Nordland Hospital Trust Bodø Norway; ^9^ Centre for Research and Education in Forensic Psychiatry and Psychology Haukeland University Hospital Bergen Norway; ^10^ Brain and Mind Centre and Central Clinical School School of Medicine, Faculty of Medicine and Health University of Sydney Sydney Australia

**Keywords:** manager; supervisor training; workplace mental health; mental health education; online intervention; knowledge; attitudes; behaviour; eHealth

## Abstract

**Background:**

Mental health has become the leading cause of sickness absence in high-income countries. Managers can play an important role in establishing mentally healthy workplaces and coordinating their organization’s response to a mentally ill worker.

**Objective:**

This pilot study aims to evaluate the feasibility, usability, and likely effectiveness of a newly developed online training program for managers called *HeadCoach*. *HeadCoach* aims to build managers’ confidence in supporting the mental health needs of staff and promote managerial behavior most likely to result in a more mentally healthy workplace.

**Methods:**

In total, 66 managers from two organizations were invited to participate in this pre-post pilot study of *HeadCoach*, which was made available to managers to complete at their own pace over a 4-week period. Data were collected at baseline and post intervention via an online research platform. The difference in mean scores for each outcome between these two time points was calculated using paired samples t tests.

**Results:**

Of all the invited managers, 59.1% (39/66) participated in the trial, with complete pre–post data available for 56.4% (22/39) of the participants. The majority of respondents reported positive engagement with the program. During the study period, managers’ knowledge regarding their role in managing mental health issues (*P*=.01) and their confidence in communicating with employees regarding mental illness (*P*<.001) significantly increased. In addition, a significant increase was observed from the baseline in managers’ self-reported actions to use strategies to prevent and decrease stress among their team members (*P*=.02).

**Conclusions:**

Although caution is needed due to the absence of a control group, preliminary results of this study suggest that *HeadCoach* could be a feasible, acceptable, and efficient method of training managers in best workplace practices to help support the mental health needs of their staff.

## Introduction

In several high-income countries, mental health conditions have become the leading cause of long-term sickness absence and occupational incapacity [1-4]. The development or persistence of mental ill health for some workers might, in part, be related to their workplace [5], a link that has now been acknowledged as a major public health concern [4]. Anxiety and mood disorders are the most common mental illnesses reported in the working population [6-8]. Although treatable and often preventable, the rates of functional impairment due to psychiatric conditions within the working age group have increased over recent decades [9], which comes at a substantial cost to individuals, their workplaces, and, eventually, the economy [4,7,10-14]. Thus, there is a growing focus on elucidating how work can affect mental health and how it can be addressed through workplace-based mental health and well-being interventions [5,15,16].

Workplace mental health programs could provide an opportunity to alter modifiable risk and protective factors for mental health and a chance to aid the identification, treatment, and rehabilitation of workers with mental health problems. Some psychosocial working conditions have been recognized as primary sources of work-related stress, which, if not managed effectively, can adversely affect workers’ well-being and productivity; these include conflicting and excessive work demands, a lack of job control, organizational failure to effectively communicate with staff, and poor collegial relationships and support [15,17]. Many of these workplace risk factors can be modified through decisions and adjustments, which managers are often in a position to make [18]. The degree to which the managers set a positive example of accepting attitudes and supportive behaviors toward the mental health of their staff can act as a protective factor for their workers. In addition, managers can react to mental ill health episodes in a way that could benefit the recovery process for workers [19]. Such strategies include, but are not limited to, facilitating regular conversations with an employee, maintaining a focus on an employee’s well-being, and developing an appropriate return to work plan if a worker is on long-term sickness absence for a mental health issue, regardless of the underlying cause [20,21]. Overall, these various preventative and responsive strategies delivered across individual, team, and organizational levels can create a mentally healthy workplace that enhances the mental health of its employees [4]. Despite the availability of best practice guidelines detailing these primary, secondary, and tertiary approaches to managing workplace mental health, managers often report uncertainty with regard to how to best support employees experiencing or at risk of mental illness [7,21].

In order to address these concerns, many organizations are introducing training for managers in how to decrease work-based mental health risk factors for their employees, support their recovery, and facilitate successful return to work following a period of sickness absence for mental ill health. There is some evidence suggesting the value of specialized training delivered to managers to promote an understanding of the mental health needs of their workers and help increase managers’ confidence in discussing mental health matters with their staff [2,3,22-24]. Further evidence supports that such manager training is effective in shifting stigmatizing attitudes regarding mental illness [24-27] and promoting the implementation of positive managerial behaviors to address mental health issues within their team [22,24,26] with an overall positive effect for manager training found across these outcomes [28]. Yet, evaluations of a selection of workplace-based mental health training programs have been unable to determine the beneficial effects on managers’ attitude toward mental illness [18] or managerial behaviors of mental health issues either reported by managers themselves or objectively by their direct reports [18,29,30]. This disparity in outcomes may be due to the selection of components included in the training. It is becoming increasingly recognized that an integrated approach is considered the best practice in workplace mental health interventions [15]. An integrated approach incorporates strategies to prevent harm, promote positive mental health, and address mental health at the workplace irrespective of the cause of the illness [15]. However, the integration of these key components has yet to become standard in manager training, resulting in the dissemination of a series of uncoordinated educational programs.

This pilot study comprises the feasibility stage of the Medical Research Council (MRC) Framework for complex interventions [31] and elucidates the development and initial testing of the delivery of a comprehensive online training intervention for managers called *HeadCoach*. *HeadCoach* integrates the components recognized as key to mental health training and aims to build managers’ confidence and ability to best support the mental health needs of the staff they supervise. The content for this online program has been derived from two separate face-to-face programs developed for managers with a focus on the mental health of their employees [22,32], which, when combined, encompass the recommended reactive and preventative components. We acknowledge that e-learning cannot offer some benefits that face-to-face contact with an educator may provide, and discussion with other participants in the course is often limited or not available. However, the modification of the delivery format from face-to-face to a mobile-responsive website offers a more flexible, time-effective, and economical means of training a large number of staff members [33]. Participants have the opportunity to schedule training around the demands of their job and may also revisit the content within a standardized learning environment, providing a better opportunity for the consolidation of the course material.

This pilot study aims to test the feasibility and usability of the *HeadCoach* program with a small group of managers prior to evaluating the program as part of a larger randomized controlled trial (RCT). The objective data on program engagement, matched with participants’ self-reported program rating scores and free text feedback, would provide information on the rates of adherence and user experience. Besides evaluating the uptake of and interaction with the program, this study aims to investigate the possible effectiveness of *HeadCoach* as a workplace mental health intervention for managers. We hypothesized that the *HeadCoach* program would help improve managers’ self-reported confidence to respond to the needs of staff experiencing mental health issues and promote their implementation of managerial techniques that would create a more mentally healthy workplace. Although the results of this study would not be capable of determining the true effectiveness of the intervention due to the absence of a control group, findings from this pre–post design may provide an initial insight into participants’ responses to and acceptability of the program and be a valuable first step in examining any impact that may be found from this type of training.

## Methods

### Development of Intervention

In accordance with the MRC Framework for complex interventions [31], we reviewed the relevant literature and a meta-analysis regarding manager training [28] to establish a theoretical basis for the development of the intervention. Following this, the *HeadCoach* online training intervention [34] was developed to improve workplace mental health by providing a flexible, easily accessible, and engaging training program for managers that informed them how to best aid the mental health needs of all their employees. Consistent with the concepts of the self-efficacy theory [35], which indicates that people are more likely to engage in particular behaviors when they feel more capable of attaining the desired behavior, this program aimed to build managers’ confidence to effectively respond to the needs of employees experiencing mental health issues and implement evidence-based managerial styles that promote a more mentally healthy workplace environment.

Previously, collaborators on this trial have conducted cluster RCTs of two different face-to-face manager training programs. The “RESPECT” Manager Training Program [22] is a 4-hour training package delivered by a clinical psychologist or a consultant psychiatrist to small groups of managers; this package combined mental health knowledge and communication training to promote more appropriate reactive responses from managers when mental health issues arise in staff they supervise. A previous RCT of this manager training showed that it resulted in changes to managers’ confidence and reactive behavior that lasted for six months at least [22]. The second existing face-to-face program, which was more preventative in its approach, aimed to improve psychosocial working conditions within an organization by changing the managers’ behavior to best promote a mentally healthy workplace [36]; the principles within this program were based on the management competencies for preventing and reducing stress at work developed by the Health and Safety Executive (HSE) in the UK [37]. *HeadCoach* was developed by combining the content from these two face-to-face programs and transforming the material into a format compatible with online delivery. Educators, designers, and IT developers with experience in creating online mental health programs were consulted for the development and appearance of the learning platform. Their primary aim was to create a functional user experience that would appeal to the target audience; this included carefully considering aspects such as the style of language in which the content would be presented in and the visual design features selected for the branding.

### Consultation Groups

As part of the modeling phase recommended when assessing complex interventions [31], we held meetings ahead of the pilot stage of the trial with representatives from various industries partnering on the evaluation of *HeadCoach*. Included in these meetings were members from the organizations’ human resources, health and well-being, and media communication teams as well as managers representing potential users of *HeadCoach*. During these sessions, we consulted participants on the relevance of the vignettes to be included in the program as well as the appropriateness of the language and style. In addition, sections of the program were also shown to determine the feasibility and usability for different industry groups. Where appropriate, adjustments were made to the content and functionality based on the feedback received from the consultation groups.

### *HeadCoach* Content

**Figure 1:**
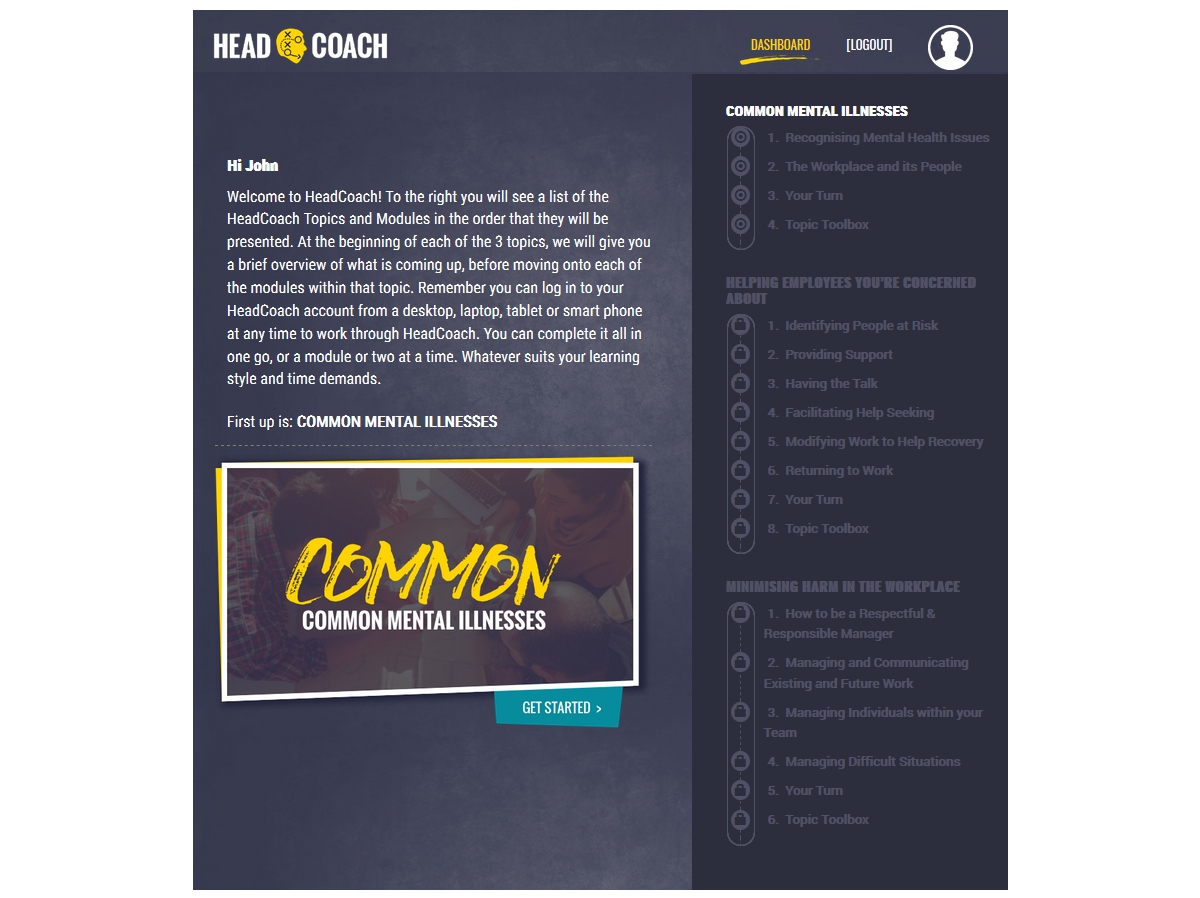
Screenshot of the *HeadCoach* dashboard.

**Figure 2:**
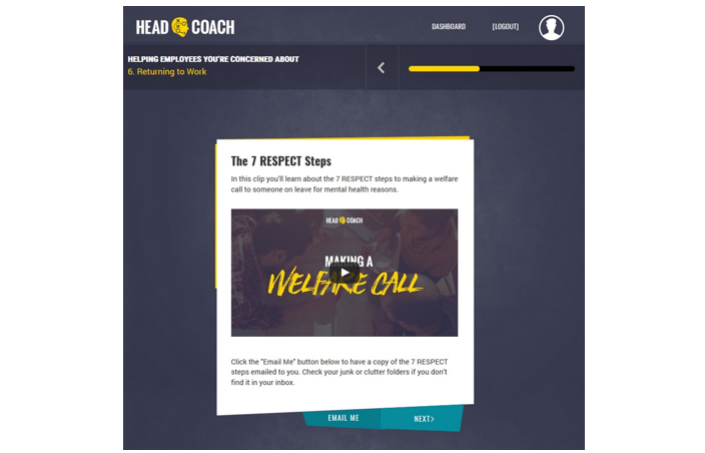
Screen shot of the landing page for a *HeadCoach* video resource.

**Figure 3:**
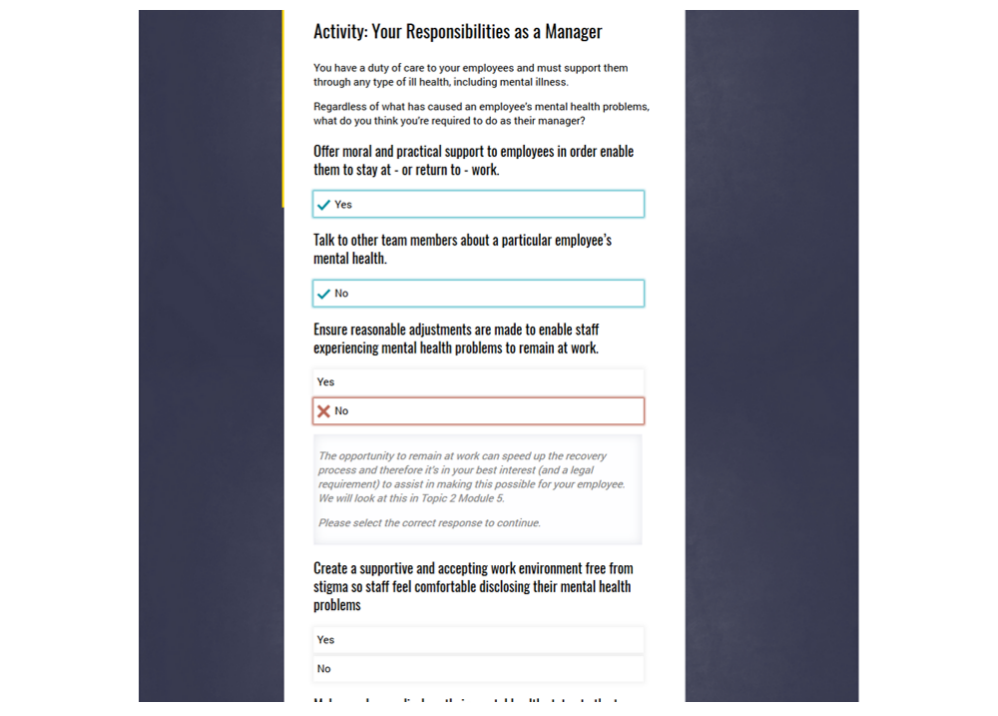
Screen shot of an interactive exercise with feedback in *HeadCoach*.

*HeadCoach* is a self-paced online intervention program that comprises three topics to be completed sequentially. Each topic contains a series of 10-minute modules featuring small sections of text, activities, short videos, practical activities, and topic summary exercises for individuals to complete. Textbox 1 presents the pilot program outline, and Figures 1-3 display the screen shots of various pages within *HeadCoach*. In addition, Supplementary File 1 details the vignettes included in the summary exercises for topics 2 and 3. The first topic, *common mental illnesses*, introduces mental health issues commonly found at the workplace. The following topic, topic 2*, how to help an employee you are concerned about*, outlines the signs managers can look out for to assist in recognizing people within their team who may be at risk of mental health issues, provides useful steps on how to initiate a conversation with employees who may be experiencing mental ill health, and discusses managerial techniques that can be implemented to support employees such as having regular catch ups with staff, creating a meaningful workplace where staff feel connected, and knowing what mental health resources are available for employees. In addition, it specifies ways to assist employees to stay at work if they wish or return to work faster following a period of sickness absence owing to mental illness. Finally, topic 3, *minimizing mental health risks at the workplace,* aims to up-skill managers with techniques useful in altering a range of workplace mental health risk factors across the individual, team, and organizational levels [21] to create a mentally healthy workplace for their employees. Strategies include how to prevent and manage work-related stress for employees by being a respectful and responsible manager, manage and communicate existing and future work for staff, tailor the management of individuals based on their individual capabilities and needs, and proactively and objectively resolve difficult situations and conflict within the team. These techniques are based on observational data [21] and management standard frameworks provided by agencies such as the HSE in the UK [37].

Participants were expected to complete *HeadCoach* in approximately 2.5 hours, although it was designed in a way that users could work through the content at a pace that suits their learning style and job demands across a 4-week period with automated weekly reminders sent to prompt program adherence. In addition, the intervention was accessed online through a mobile-responsive website using a desktop, laptop, tablet, or mobile phone.

### Study Design

This pilot study was primarily conducted to test the feasibility and usability of *HeadCoach* and investigate, through self-reported responses, early evidence of its possible effectiveness in altering managers’ level of confidence regarding dealing with mental health matters at the workplace. In order to maximize the opportunity to test the functionality of the product and receive substantial feedback on the processes and quality of training, all participants were allocated to receive the intervention. Participants comprised managers employed by one of the two industry partners collaborating on the pilot study. *HeadCoach* was delivered to managers individually through their usual computer, tablet, or Web-based mobile phone. Through an online form, the managers provided informed consent to participate in the study.

### Recruitment

Two organizations in Australia volunteered to participate in the pilot evaluation of *HeadCoach.* One organization was an equipment and machinery hire company servicing Australia across metropolitan, regional, and remote areas; managers in this organization supervised branches and employees across one or more offices or regions. The second organization provided a statewide fire and rescue service; managers in this organization who were eligible for this pilot study were employed at the duty commander or station officer level and were responsible for one or several different fire stations. For both organizations, the managerial level identified as relevant for this pilot study comprised supervisors who were the primary contact for staff members regarding periods of sickness absence or when workplace issues arose.

**The course outline for the pilot version of *HeadCoach*.**

**Topic 1: Common Mental Illnesses**

- Module 1: Recognizing mental health issues
- Module 2: The workplace and its people
- Module 3: Topic summary exercises

**Topic 2: How to help an employee you are concerned about**

- Module 1: Identifying people at risk
- Module 2: Providing support
- Module 3: Having the talk
- Module 4: Facilitating help seeking
- Module 5: Modifying work to help recovery
- Module 6: Returning to work
- Module 7: Topic summary exercises

**Topic 3: Minimizing mental health risks in the workplace**

- Module 1: How to be a respectful and responsible manager
- Module 2: Managing and communicating existing and future work
- Module 3: Managing individuals within a team
- Module 4: Managing difficult emotions
- Module 5: Topic summary exercises

Participants met the inclusion criteria if they were currently supervising a team of ≥3 employees, aged ≥18 years, and currently residing in Australia with a good level of English comprehension. We contacted 66 managers via email who were identified by their employer as those fulfilling the inclusion criteria. The email described the purpose of the research, outlined what would be involved, and contained a link to register for the pilot study. In addition, the email emphasized that although participation in the trial was supported by their employer, it was entirely voluntary, and their level of involvement in the study would remain confidential from their employer.

### Procedure

**Figure 4:**
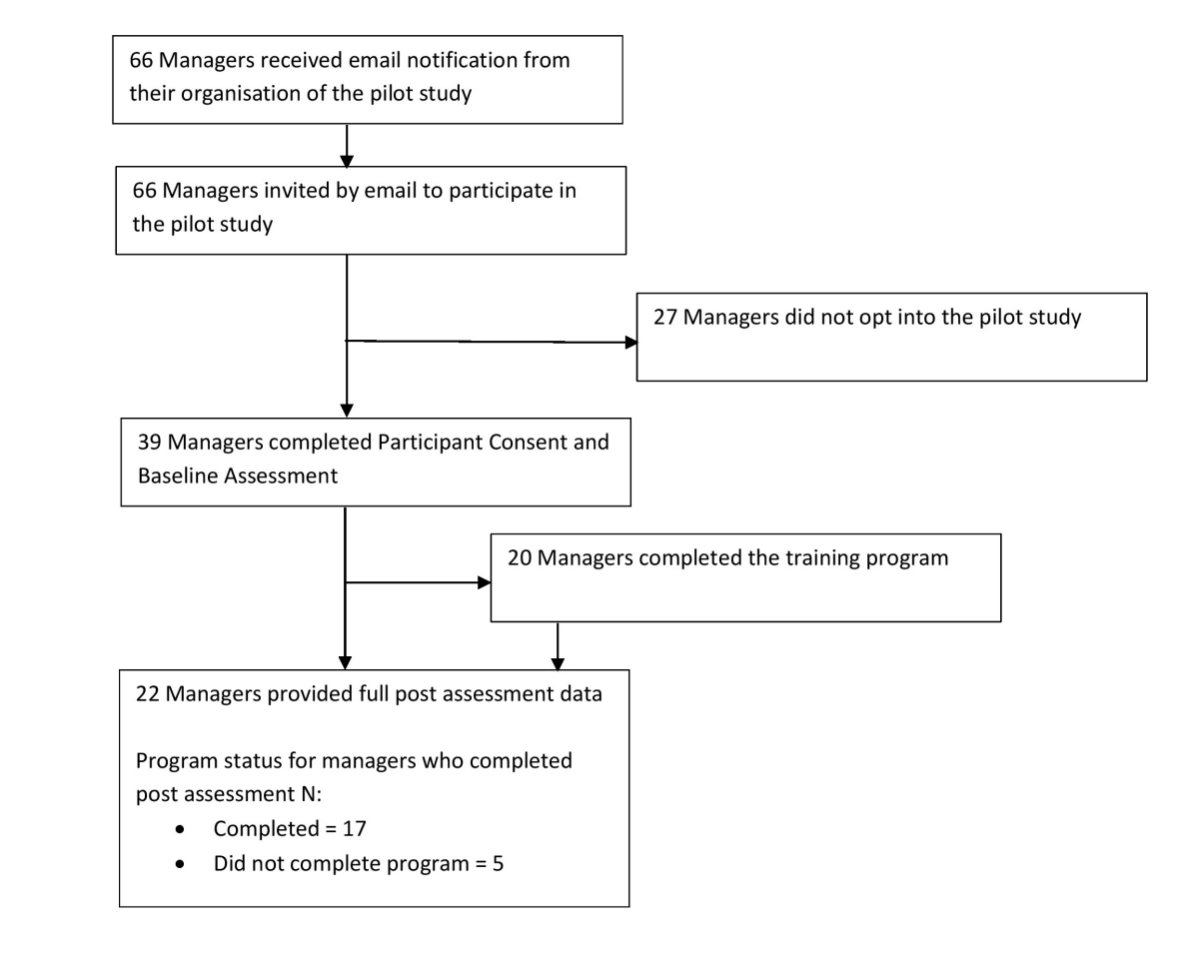
*HeadCoach* pilot study procedure flow.

The trial procedure and stages of assessment for participants are outlined in Figure 4. All eligible to participate in the study received an information email from the researchers, inviting them to participate in the study. A hyperlink provided in the email directed them to the *HeadCoach* study website, which detailed further information about the program, the online consent form, and the registration page. Once registration was complete, the online baseline assessment was made available, following the completion of which participants were able to commence the online *HeadCoach* manager training program. The design of the program allowed managers to work through the program at their own pace over a 4-week period. Emails were automatically distributed weekly from sign up until participants had completed the program; these emails informed participants of the time remaining to complete the program and served as a reminder to revisit their account to continue working through the program.

Follow-up questionnaires were disseminated 4 weeks after completion of the baseline questionnaire. This 4-week period comprised the training period allocated to managers to complete *HeadCoach*. If all the components of the online program were completed earlier in the 4-week training period, the participant was invited to complete the follow-up questionnaire at that time point. This approach was selected to reduce nonresponse.

### Data Collection

The baseline and postintervention data were collected electronically via the research platform that hosted the *HeadCoach* program, allowing a streamlined process between completing the *HeadCoach* program and completing questionnaires. In the case of nonresponse, two reminders were automatically sent across the subsequent 10 days via the research platform. On the completion of the 4-week postintervention questionnaire, the *HeadCoach* content was made available again for participants within their account for their reference.

#### Measures of Usability and Feasibility

At the 4-week postintervention follow-up, participants were asked whether “This online course was engaging and interesting?” with response options ranging from *strongly disagree* to *strongly agree*. In addition, questions were asked evaluating the ease of navigating the program and finding information, whether the course fulfilled their expectations, and how likely would they recommend the program to their colleagues. Participants were also provided with an opportunity to provide detailed feedback through additional free text questions, including suggestions on what should be included in future versions. Finally, we obtained data regarding completion rates, including the duration of time taken to complete each module, from the research platform database.

#### Measures of Effectiveness

We measured managers’ confidence in managing mental health issues and promoting a mentally healthy workplace using a modified version of the previously described supervisor scales [19]. Managers were asked to indicate their current level of confidence on a 5-point Likert scale with options ranging from *not at all confident* to *extremely confident*. Online Supplementary File 2 details the questions used for this measure. Knowledge about common mental health was assessed using the first 6 questions of the Mental Health Knowledge Schedule (MAKS) [38]. Nonstigmatizing attitudes toward mental illness was assessed using a modified version of previously published measures of personal stigma [39-41]. Comprehension of their role as a manager in dealing with mental health in the workplace was assessed using a questionnaire developed in accordance with the core competences outlined in a report detailing managers’ role in supporting return to work after ill health [20]. The managers’ application of managerial techniques that promote a mentally healthy workplace was assessed using an adapted version of the HSE Management Standards Indicator Tool [17]. We used a 5-point Likert scale with response options ranging from *strongly disagree* to *strongly agree* for questions such as “I provide regular opportunities for my team to speak one to one” and “I give employees the right level of job responsibility.” Online Supplementary File 2 provides the remaining items. All questions were asked at the baseline and at the 4-week postintervention assessment. We converted the participants’ scores to a percentage of the maximum possible score prior to data analysis.

We collected demographic information including age, gender, job role and length of service in the role, number of employees currently supervising, and previous mental health training.

### Statistical Analysis

Participants’ use of the training program was described in terms of the total time and number of modules completed. Descriptive statistics were used to demonstrate participants’ responses to the questions on the usability and acceptability of *HeadCoach*. We assessed the differences in the mean percentage scores of each of the outcomes between baseline and post intervention collected at the 4-week follow-up using paired samples *t* tests. The parameters included managers’ confidence in managing mental health issues within the workplace, level of mental health literacy, nonstigmatizing attitude toward mental illness, understanding of their role in managing mental health issues within their team, and application of managerial techniques that promote a mentally healthy workplace. All statistical analyses were conducted using SPSS version 23.

## Results

### Demographics

In this study, all managers were enrolled during November 2016 and December 2016 with the postintervention data collected during December 2016 and January 2017. Among the 66 managers who were invited to participate in the *HeadCoach* pilot study, 59.1% (39/66) registered and completed the baseline assessment. All managers were assigned to receive the intervention. Table 1 outlines the demographics of the study sample, including baseline characteristics of the entire sample and participants included in the final analysis.

### Usability and Feasibility of *HeadCoach*

Over half of the study sample (51.3%; 20/39) completed all 15 modules of the *HeadCoach* program. The time taken to complete the program ranged from 43 minutes to 2 hours 53 minutes. We obtained feedback on the program through the postquestionnaire. Of those who responded, 77% (17/22) found *HeadCoach* engaging and interesting, 86% (19/22) agreed it was easy to find the information needed, 73% (16/22) reported that the program fulfilled their expectations, 91% (20/22) considered it useful, and 86% (19/22) considered it worth recommending to a friend.

**Table 1:** Demographics as reported at the baseline.

Demographic			Total sample (n=39)		Sample with pre- and postintervention data available (n=22)
Age (years), mean (SD)			45.03 (8.74)		47.86 (8.44)
**Gender, n (%)**					
	Male	34 (87.2)		20 (90.9)	
	Female	5 (12.8)		2 (9.1)	
**Industry, n (%)**					
	Building/Construction	12 (30.8)		5 (22.7)	
	Emergency Services	27 (69.2)		17 (77.3)	
**Years at current employer, n (%)**					
	<1	3 (7.7)		2 (9.1)	
	1-5	5 (12.8)		2 (9.1)	
	5-10	5 (12.8)		2 (9.1)	
	10-15	4 (10.3)		2 (9.1)	
	>15	22 (56.4)		14 (63.6)	
**Years in this level or above, n (%)**					
	<1	6 (15.4)		2 (9.1)	
	1-5	9 (23.1)		4 (18.4)	
	5-10	14 (35.9)		10 (45.5)	
	10-15	6 (15.4)		4 (18.2)	
	>15	4 (10.3)		2 (9.1)	
**Modules completed, n (%)**					
	0	7 (17.9%)		1 (4.5)	
	1-7	8 (20.5%)		1 (4.5)	
	8-14	4 (10.3%)		3 (13.6)	
	15 (Completed program)	20 (51.3%)		17 (77.3)	
	Mean (SD)	9.08 (6.62)		12.95 (4.37)	

**Figure 5:**
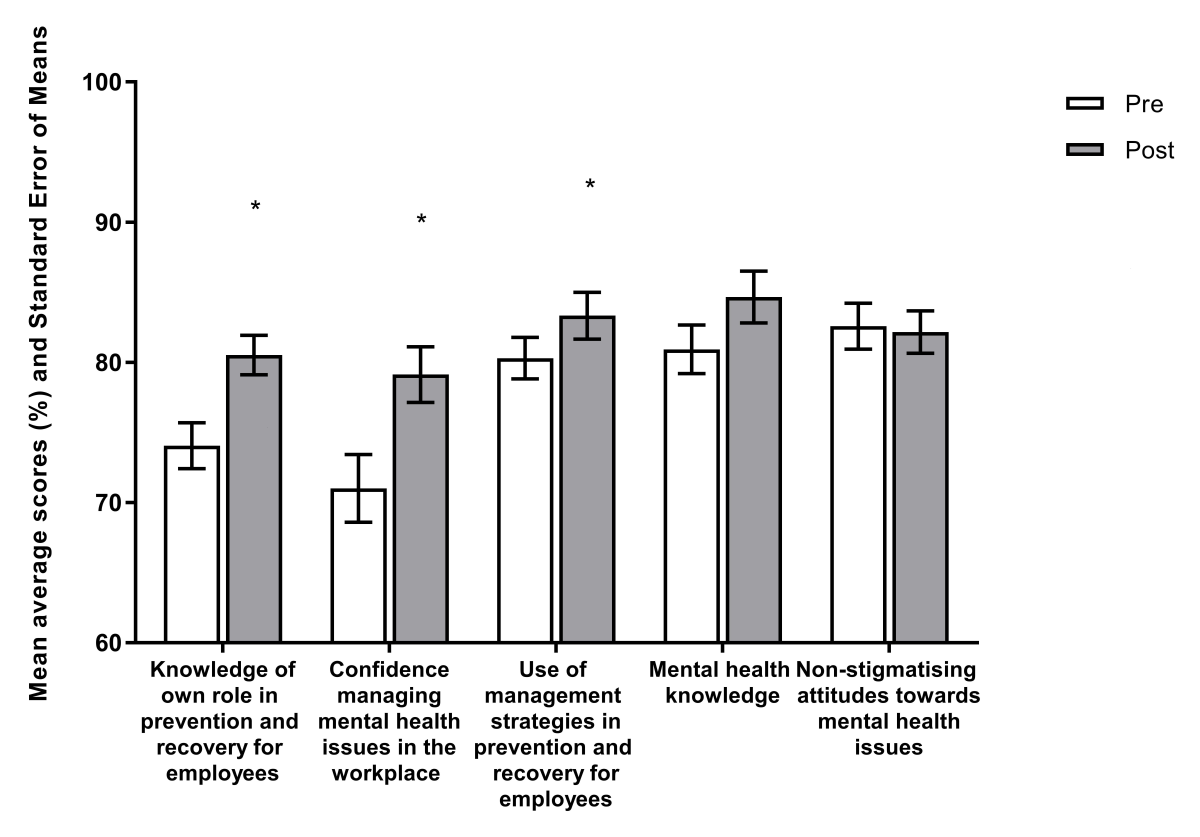
Baseline and 4-week post intervention mean average scores (%) and standard error of means for outcomes.

The qualitative feedback received post evaluation was primarily positive. All participants highly valued the practicality of the information and the format it was presented in. Examples of comments reflecting these views included “This topic was useful in giving logical and practical guidance on early intervention and strategies to minimize harm occurring in the workplace,” “Good tips on initiating conversations and advice on where to go for resources,” “It was great to get some practical examples and information that could be used in my workplace,” and “Very well explained and easy to understand.” In addition, comments around improved confidence to manage workplace mental health issues included “I found that it [*HeadCoach*] consolidated what I had covered previously in training and in doing so gave me more confidence to address an issue should one arise” and “[*HeadCoach*] gave me a bit more confidence in my approach.” The key negative feedback regarding the content related to the level of detail included about common mental illnesses (eg, “Having a family history with mental illness, I hoped this course may be more informative, it tends to be a little superficial or shallow in details of symptoms to look out for”). This feedback prompted the implementation of more detailed information into the subsequent version of *HeadCoach* with the inclusion of direct access to additional online resources.

### Effectiveness of *HeadCoach*

Figure 5 displays that following their use of *HeadCoach*, managers exhibited significantly higher levels of self-reported confidence in communicating with employees regarding mental illness (*t*_22_=4.180; *P*<.001) and actions to employ managerial strategies to prevent and reduce stress among their team (*t*_21_=2.468; *P*=.02). In addition, we observed a significant increase in managers’ knowledge regarding their role in managing mental health issues (*t*_23_=2.881; *P*=.01). At the follow-up, no significant change was noted in nonstigmatizing attitudes toward mental illness (*t*_23_=–0.268; *P*=.80), whereas an increase in the levels of mental health knowledge fell just short of statistical significance (*t*_24_=1.987; *P*=.06).

## Discussion

### Principal Findings

This pilot study assessed the feasibility, usability, and likely effectiveness of *HeadCoach,* a newly developed online training intervention for workplace managers regarding the mental health needs of employees reporting directly to them. To the best of our knowledge, *HeadCoach* is the first educational program for managers, which is delivered entirely online and provides an integrated program of preventative and reactive content consistent with recently recommended best practice frameworks for workplace mental health [4,15].

In this pilot study, we collected relevant data to assess the feasibility of recruitment and participation processes and the usability of the program through self-reported measures and also objectively, as provided through the online research platform. This online platform has the potential to provide valuable information regarding levels of adherence and engagement [42]. The findings suggest that for *HeadCoach,* the online recruitment and registration processes, as well as methods for gathering data and delivering course material as operated through the current online research platform, were a practical and acceptable means for the dissemination and collection of various forms of information.

This study also suggests that *HeadCoach* correlated with significant increases in the managers’ knowledge of their role in managing mental health issues among their staff, their confidence to do so, and their application of management strategies to promote a mentally healthy workplace. These changes in manager outcomes correspond to prior RCTs evaluating face-to-face training for managers promoting both reactive and preventative strategies to manage mental health issues within their team [22,26]. Although this study only evaluated the effect of *HeadCoach* with a pre–post design, the preliminary findings hold prominence because they suggest that e-learning options could replicate the positive outcomes previously reported with similar face-to-face manager training. This is an encouraging prospect because, if proven, it would allow organizations a practical means of training a large number of managerial staff about mental health issues with minimal time away from their jobs. Online training is a flexible and convenient format of learning because it can be structured to fit around other responsibilities and deadlines that managers are required to meet in their daily role. Although further evaluation through an RCT is warranted, these findings suggest the potential of *HeadCoach* to provide feasible, acceptable, and effective workplace mental health training for managers.

### Limitations

Despite the novelty of our findings, there are a number of important limitations. As mentioned previously, the sample size was small with just over half of participants (51.3%; 20/39) completing the online intervention, and 56.4% (22/39) of the sample providing pre- and postintervention data. Although this dropout rate is typical in studies on online training [42,43] and survey responses [44], a higher response rate for both the adherence and follow-up would be ideal to minimize any potential bias caused by nonresponse. Besides the significance of personalized reminders [44], alternative engagement strategies may need to be adopted to help prompt adherence to the various stages of the trial, such as varying the messages contained within the email reminders and clearly conveying the purpose of participation [45]. In addition, strategies to maximize the response rates to the final questionnaire, such as the inclusion of a prize draw, may be valuable to increase the odds of participants responding [45].

The generalizability of results from this pilot study is limited by the opt-in approach to recruitment. The sample of managers who agreed to participate and who completed the multiple components of the trial might have held a pre-existing awareness of mental health issues with an interest to further develop their skills to best support their employees. This may explain the lack of substantial change found in managers’ mental health knowledge because the sample may have already been well-informed of mental health issues and, therefore, a ceiling effect may have affected the outcomes of this study. Alternatively, the lack of marked effect may have been due to the lack of power, and with a larger sample size, a substantial change may have been observed for these outcomes. The generalizability of results may be limited by the sample of managers participating in this pilot study; the managers were from two specific industries, and nearly two-thirds (63.6%; 14/22) had been with their current employer for >15 years. An investigation within a variety of organizational contexts with a more representative sample is warranted before drawing conclusions about the feasibility and possible effects of this program for managers outside the building construction and emergency services industries.

The absence of a control group further limits the conclusions that could be drawn regarding the efficacy of the intervention because other factors explaining the observed change cannot be ruled out. Another limitation was the short follow-up period included in this trial. Although we observed a marked change postintervention, conclusions about the persistence of intervention effects over time cannot be drawn because of the lack of an extended follow-up. The short evaluation period included in this study may have also been insufficient to allow managers the opportunity to display a change in attitude within their work environment, yet with the inclusion of a longer follow-up, a change in nonstigmatizing attitudes could have been observed. A prolonged evaluation period would allow for employee-level data to be obtained for assessing the impact of manager training as perceived by the employees, providing valuable objective information about the flow on effects of manager training for the staff they supervise. The final potential limitation to note is the collection of self-reported data*,* especially regarding confidence levels. Participants may be more likely to report their levels of confidence or behavior favorably, although the use of identical questions at both time points and the anonymity of an online survey should have decreased this risk.

### Conclusions

Although testing in a more comprehensive study with the inclusion of a control group is required to demonstrate a true effect*,* the preliminary findings from this pilot study suggest that *HeadCoach* may provide a practical and efficient method of training managers in best workplace mental health practices. Given the key role managers play in the promotion of mental health and well-being within their teams, there is great potential for this type of training to improve the support provided to employees for their mental health needs in the future.

## Appendix

Multimedia Appendix 1 Supplementary File 1 Vignettes for Summary Exercises.

Multimedia Appendix 2 Supplementary File 2 Questionnaire.

## Abbreviations

GEM: Guided e-learning for managers
MHAT: Mental health awareness training
MRC: Medical Research Council
RCT: Randomized controlled trial
HSE: Health and Safety Executive
