# Supplementary material for: A New Online Mental Health Training Program for Workplace Managers: Pre-Post Pilot Study Assessing Feasibility, Usability, and Possible Effectiveness
Source: JMIR Ment Health. 2018 Jul 3;5(3):e10517. doi: 10.2196/10517 (PMC6053610; doi:10.2196/10517)
Supplement: Multimedia Appendix 1 [file mental_v5i3e10517_app1.pdf]

Online Supplementary File 1

***HeadCoach* Pilot Study Topic Summary Exercises**

Topic Exercise 2: HELPING EMPLOYEES YOU ARE CONCERNED ABOUT

Your Turn

Now it's your turn to put into practice the knowledge you've just learned in Topic 2. Work your way through the following scenarios to reveal the best management approach to the situation. If you select the incorrect choice, you will be given the opportunity to select the correct one before proceeding to the next.

Exercise 2.1.

- John is one of the guys you manage. John has been taking more sick days than usual and when he is at work he is struggling to stay a top of his tasks. What do you do?

**Option**	**Feedback**
Let him know it's time for his performance review?	Before conducting a performance review, it's a good idea to check if anything is happening within the workplace or in John's personal life that might be affecting his work.<br>Please select the correct response to continue.
Email him to ask him what is happening?	Emails can be easily misinterpreted so it is best to avoid.<br>Please select the correct response to continue.
Organise a meeting to see if he's ok?	Yes! It's best to talk face to face.

- John turns up at the scheduled time but asks what the purpose of the meeting. You:

**Option**	**Feedback**
Start out by asking him about the diminished quality of work he's producing?	This approach would not be conducive to helping John feel safe to disclose his situation to you and therefore he would not get the help he needs right now.

	Please select the correct response to continue.
Enquire how things are at work and home?	Yes! This positive meeting is to help you understand what is affecting John's work, and to start the process of John getting some help
Tell him it seems like something is up and he needs some time off to regroup?	Time off might not be what John needs right now.<br><br>Please select the correct response to continue.

- John says everything is fine. Do you

**Option**	**Feedback**
Tell John you'll organise a performance review?	The response John gave you isn't enough for you to conclude that he really is fine<br><br>Please select the correct response to continue.
You mention how John is usually on top of his game but that you have noticed things are a bit different lately?	Yes this shows that you appreciate the work John usually delivers but that you have noticed something might be happening.
Talk about a time when you were struggling to cope with work and life?	This meeting is about John. Your experience may not be relevant or appropriate to discuss.<br><br>Please select the correct response to continue.

.

- John discloses he's been feeling very anxious lately, having some panic attacks and having a lot of trouble sleeping. Do you

**Option**	**Feedback**
Tell him that if he exercised more it would help?	Exercise is beneficial for mental health, but offering solutions to 'fix' John is not your role.<br><br>Please select the correct response to continue.
Ask him if he has seen someone who could help him and if he would like some time off to attend	Yes! This shows concern and support for your worker.

appointments?	
Give him the number of the welfare officer at work?	This would be helpful to have but it would be good to have some more information about John's situation before giving him these details.<br><br>Please select the correct response to continue.

Topic Exercise 2: HELPING EMPLOYEES YOU ARE CONCERNED ABOUT

Exercise 2.2.

- Your employee Taylor has been much more withdrawn than usual. You saw her in the lunch room crying but didn't stop as you were rushing to a meeting so planned to speak with her the next day. However, in the morning you got an email with a doctor's certificate stating Taylor was unfit for work for 2 months. **What do you do?**

**Option**	**Feedback**
You are satisfied that Taylor is receiving medical help and therefore she won't need your help.	*Support for your staff shouldn't stop because they are on sick leave. You have an important role to play in keeping lines of communication open so when she is well enough and ready to return there are less barriers to do so.*<br>*Please select the correct response to continue.*
Email her to ask what's happening?	*Emails can be misinterpreted and feel a bit impersonal. It's not guaranteed she will receive the email as she may not be checking work emails.*<br><br>*Please select the correct response to continue.*
Send a text message expressing concern. Ask if it's to call her to chat, and when?	*Yes. When depression is very severe responding to texts or making a phone call can feel overwhelming. Your text lets Taylor know you are thinking of her and lets her know you will be calling (do call in a few days even if she doesn't respond for the some reason)*

- Taylor's partner calls you back to explain that Taylor is in hospital for treatment of depression. Although Taylor isn't up to speaking to anyone at the moment, her partner asks if you could make contact in a week. And to keep this confidential. **What do you do?**

Option	Feedback
Without specifying when, you tell Taylor's partner you will call back.	*Schedule the call into your calendar like you would a meeting. This will help you remember to do it. Support for Taylor shouldn't stop because she's now off on sick leave.*<br>*Please select the correct response to continue.*
Reassure Taylor's partner about privacy and confidentiality and together you decide on a day and time for you to call back.	*Yes. You still need to keep contact and keeping Taylor or Taylor's family involved in the decision making around this is important. It will be hard to keep this information confidential but it is important to do so. Later down the track you might try and support Taylor to disclose this to her colleagues but right now she needs your reassurance.*
You explain to Taylor's partner just how hard it's going to be for you to reallocate Taylor's duties without giving some reason to the team.	*You are applying a lot of pressure on Taylor's partner which they don't need right now. Taylor's privacy and confidentiality around her mental health treatment needs to be maintained. If you are concerned about how you can explain this to her colleagues get support from HR or Manager Assist*<br>*Please select the correct response to continue.*

- 5 weeks on and you are regularly in contact with Taylor via text messages and phone. Taylor tells you that her Psychiatrist has advised her to start thinking about returning to work. She indicates that she's really worried about coming back in case she "fails again and will let everyone down". **How do you respond?**

Option	Feedback
Agree with Taylor that you'd rather see her come back when he feels she's 100% fit. You make a time to call her again in 2 weeks like you've been doing.	*Getting back to work has been shown to help people recover from an episode of mental illness like depression faster than being out of work. Start talking through options.*<br>*Please select the correct response to continue.*
Reassure Taylor that she didn't 'fail' and show support for the psychiatrist's confidence in the progress Taylor	*Yes. Taylor doesn't need to be 100% better before starting back at work and that getting back to work has been shown to help people recover from an episode of mental illness like depression.*

has made. Explain how after a period of time off work it's been shown that a graded return helps people readjust to work, reduces stress and can speed up recovery.	
Ask Taylor when she thinks she'll be ready to return.	*Respecting Taylor's concerns and getting her input is important in this process, however, the answer to this question may not be known, and may only delay Taylor's rehabilitation.*<br>*Please select the correct response to continue.*

- Taylor gives you permission to speak with her psychiatrist who explains that Taylor is responding well to medication but suggests she returns to work 2 days a week initially and starts a bit later in the day due to ongoing fatigue that is worse in the mornings. **What do you do next?**

**Option**	**Feedback**
You struggle to work out how this arrangement can be made and sustained so make a time to speak with HR to see what other supports are available for Taylor and yourself.	*Yes. Supporting others in this area can be difficult and it is important that you seek advice and support for yourself when needed.*
You explain to Taylor you need her to work at least 4 days/week but as a compromise you are happy to allow her to start an hour later than normal.	*Ignoring the psychiatrist's recommendation isn't conducive to helping Taylor prepare for return to work. Arrange a face to face meeting with Taylor, you manager and HR to come up with an arrangement that works for everyone.*<br>*Please select the correct response to continue.*
Even though you are unsure how this will work, you agree to this arrangement.	*It is best to iron out any concerns before finalising the arrangement. This will prevent longer term problems which could hinder Taylor's rehabilitation and successful return to full time work.*<br>*Please select the correct response to continue.*

Topic Exercise 3: MINIMISING HARM IN THE WORKPLACE

Your Turn

Now it's your turn to put into practice the knowledge you've just learned in Topic 3. Work your way through the following scenarios to reveal the best management approach to the situation. If you select the incorrect choice, you will be given the opportunity to select the correct one before proceeding to the next.

Exercise 3.1:

- You've been managing Alan for a few years now. In your mind, he's a solid performer – he could even be ready to step up to a new role. You consult your Skill/Will model and decide Alan should mentor some of your newer members to prepare for his possible promotion to team leader. To your surprise, he's not happy about the decision. **Do you…**

Options	Feedback
Tell Alan you're surprised he doesn't want to take on the additional duties in preparation for his next career move as he would make a great team leader.	*It's great that you see Alan as a top performer and want to develop him, but it's also important not to let your own feelings influence your judgement. How much do you know about his career aspirations? It's better to have a conversation with him first and agree together what his next move could be.*
Realise you'd just assumed Alan would want to be eventually be a team leader. You organise a meeting with him to find out about his career goals and how you can help him achieve them.	*Yes! It's great that you want to support his development, but it's important to take the time to understand Alan's career aspirations and help him achieve this.*
Start telling Alan about your own career experience and exactly what he needs to do to get ahead in the role.	*It's better to ask him about where he sees himself going in the role, rather than telling him he should do (even if you're trying to be helpful).*

- Alan's expressed interest in expanding his skills and spending sometimes in the sales department, which you also oversee. This morning you received a call from a customer who's been sent the wrong invoice from Alan for the second time in a row. They're clearly not happy. **What do you do?**

Options	Feedback
Email Alan to let him know you're not happy, but you'll let it slide this time. He's new in this role after all – hopefully he'll get the hang of things.	*In this situation, it would be better to understand why these mistakes are happening. A conversation or phone call is always preferable to emails, which can be misunderstood.*
Call Alan up straight away to let him know he can't afford these kind of mistakes. If it happens again you'll give him an official performance warning and be forced to move him back to his original role.	*Whilst it's normal to feel frustrated in a situation like this, it's important to have the conversation with Alan once you're in a calmer state.*
Arrange to talk with Alan to see how he's managing the new role and what you can do to get his performance where it needs to be - easing into a discussion about the customer's complaint.	*Yes - this is a respectful and responsible way to address the situation.*

- You have a conversation with Alan the next day as planned, explaining the nature of the complaint and the impact it could have on the business. Whilst defensive at first, Alan eventually indicates he's feeling in over his head with the new role. Hearing him talk, you now realise that there is a real skill gap here in terms of his understanding of the administrative side of the job. **What do you do?**

Options	Feedback
Offer to stay back late with Alan the next few nights to go through the administrative side of the job and get him back on top of things.	*It's great that you're offering to help Alan out with some on-the-job development, but ideally this should occur during normal work hours. Staying after work may be perceived as punitive action and cause additional stress for Alan. You also need to be considerate of his work- life balance - and yours too!*
Discuss various options to ensure Alan has the support he needs to get up to speed, including partnering him up with a more experienced team member who you know is a good on-the-job coach.	*Yes! Great response. As a manager, it's critical that you provide on-the-job support to effectively balance the demands of the job.*
Consider taking Alan out of the role until he's had more time to get his head around things. Perhaps he needs to attend some off-site training?	*Whilst training could be a good way to ensure Alan has the skills he needs to manage the increased demands of the job, taking him out of his role will likely disempower Alan and make him feel like he's being demoted.*
