# Supplementary material for: A New Online Mental Health Training Program for Workplace Managers: Pre-Post Pilot Study Assessing Feasibility, Usability, and Possible Effectiveness
Source: JMIR Ment Health. 2018 Jul 3;5(3):e10517. doi: 10.2196/10517 (PMC6053610; doi:10.2196/10517)
Supplement: Multimedia Appendix 2 [file mental_v5i3e10517_app2.pdf]

Online Supplementary File 2

***HeadCoach* Pilot Study Scales Measuring Manager Confidence and Managerial Techniques**

Section 4 (Supervisor Confidence)

Please indicate your current level of CONFIDENCE with the following:

		Not at all confident	Not really confident	Slightly confident	Confident	Extremely confident
1.	Initiating contact with staff on sickness absence leave that you believe might be due to mental illness	1	2	3	4	5
2.	Discussing rehabilitation/return to work plans with the staff member on sickness absence	1	2	3	4	5
3.	Identifying barriers preventing a staff member returning to work and coming up with solutions to those barriers	1	2	3	4	5
4.	Initiating a conversation with a staff member who is not on sickness absence but who you think may be suffering from mental illness	1	2	3	4	5
5.	Initiating a conversation with staff members who have recently been exposed to a potentially traumatic event and enquiring about their well-being	1	2	3	4	5
6.	Creating a work environment that prevents and reduces stress within my team	1	2	3	4	5

Section 5 (HSE for Managers)

Below are some statements about managerial strategies to prevent and reduce stress at work. Please indicate your level of agreement with each statement. Please answer these questions as honestly as possible (remember your answers for all questions will remain anonymous).

		Strongly disagree	Disagree	Slightly agree	Agree	Strongly agree
1.	I do what I say I will do	1	2	3	4	5
2.	I don’t pass on my stress to my team	1	2	3	4	5
3.	I give more positive than negative feedback	1	2	3	4	5
4.	I monitor my team’s workload on an ongoing basis	1	2	3	4	5
5.	I give employees the right level of job responsibility	1	2	3	4	5
6.	I provide regular opportunities for my team to speak one to one	1	2	3	4	5
7.	I try to see things from my team members’ point of view	1	2	3	4	5
8.	I deal objectively with employee conflicts	1	2	3	4	5
9.	I seek advice from other managers when necessary	1	2	3	4	5
